# Diagnostic Utility of ANG in Coronary Heart Disease Complicating Chronic Heart Failure: A Cross-Sectional Study

**DOI:** 10.1155/2016/2740826

**Published:** 2016-10-31

**Authors:** Peng Yu, Ming Liu, Xue Yang, Ying Yu, Ji Zhao, Lei Zhang, Rui Tong, Hong Jiang, Yunzeng Zou, Junbo Ge

**Affiliations:** Shanghai Institute of Cardiovascular Diseases, Zhongshan Hospital, Shanghai Medical College of Fudan University, Shanghai 200032, China

## Abstract

Angiogenin (ANG) has been shown to be elevated in several cardiovascular diseases. To detect its levels and diagnostic capacity in coronary heart disease (CHD) patients complicating chronic heart failure (CHF), we performed this cross-sectional study and enrolled 616 CHD patients and 53 healthy controls. According to complicating CHF or not, the patients were divided into CHF group (*n* = 203) and CHD disease controls (*n* = 413), in which the CHF group was subdivided as heart failure with reduced ejection fraction (HFrEF) group or heart failure with preserved ejection fraction (HFpEF) group on the basis of left ventricular ejection fraction (LVEF), or as different NYHA class group. Their plasma ANG levels were detected using enzyme-linked immunosorbent assay (ELISA). Plasma ANG was 342.8 (IQR [273.9,432.9]), 304.5 (IQR [254.0,370.5]), and 279.7 (IQR [214.4,344.0]) ng/mL in the CHF group, CHD disease controls, and healthy controls, respectively, significantly higher in the CHF group compared with the others. Furthermore, among CHF group, ANG is dramatically higher in the HFrEF patients compared with the HFpEF patients. As for the diagnostic capacity of ANG, the area under the receiver operating characteristic curve was 0.71 (95% CI 0.63–0.78). We concluded that plasma ANG is elevated in CHD complicating CHF patients and may be a moderate discriminator of CHF from CHD or the healthy.

## 1. Introduction

Chronic heart failure (CHF), as the terminal stage of all cardiovascular diseases, is a major and growing public health problem worldwide. Common causes of heart failure are coronary artery disease (CHD), high blood pressure, and diabetes [1, 2]. With the improvement of management for coronary heart diseases, CHD complicating CHF is increasing. To distinguish CHD patients complicating CHF from the CHD only one remains a problem. No single diagnostic test for CHF exists which is largely a clinical diagnosis based on a careful history and physical examination; N-terminal pro-B-type natriuretic peptide (NT-proBNP) is a prevailing test for the heart failure diagnosis and prognosis, but it is highly dynamic in cardiac ischemia [3]. Therefore, it is urgent to find new biomarkers for CHF diagnosis.

ANG, one of the most potent angiogenic factors, appears to be elevated in various cardiovascular disorders (e.g., myocardial infarction [4], stable coronary artery disease [5], and heart failure [6, 7]). Some research has suggested that, in the heart remodelling process, angiogenesis plays an important role [6, 8–10]. Thus, ANG may be a potential diagnosis biomarker for the CHF, which is coupled with cardiac remodelling.

Previously, we have demonstrated ANG may be a biomarker for the diagnosis and prognosis of HFpEF [7]. Here we mainly detected the difference of plasma ANG between the CHD patients complicating CHF or not, explore its levels in different CHF subgroup, and further evaluate its diagnostic capacity for CHF.

## 2. Methods

### 2.1. Study Patients and Controls

Patients consecutively admitted in Zhongshan Hospital from December 2013 to November 2014 were eligibly enrolled to this cross-sectional study if they were (1) male or female age ≥ 40 years; (2) diagnosed with CHD, complicating CHF symptoms or not; (3) willing to provide written informed consent. Patients were excluded if they had (1) clinically acute myocardial infarction [11] (if they met any of the following diagnosis criteria: (1) plasma cardiac troponin T (cTnT) > 0.12 ng/mL, (2) new or presumed new significant ST-segment-T wave (ST–T) changes or new left bundle branch block (LBBB), (3) development of pathological Q waves in the ECG, and (4) identification of an intracoronary thrombus by angiography); (2) severe obstruction with hypertrophic obstructive cardiomyopathy, or dilated cardiomyopathy; (3) severe diseases such as tumor and HIV infection. The definition of CHF is based on the guidelines from the American College of Cardiology/American Heart Association [1]. Healthy subjects were recruited from the Department of Health Examination in our hospital.

Fasting whole blood was obtained with prechilled Na-EDTA tubes (BD, New Jersey, USA) from each participant and the plasma was stored at −80°C until assay. All subjects signed an informed written consent to participate in the study that was approved by Ethical Committee of Zhongshan Hospital, Fudan University, China, which is according to the principles stated in the Declaration of Helsinki.

### 2.2. Laboratory Tests and Plasma ANG Detection

Plasma ANG levels were tested in all subjects by enzyme-linked immunosorbent assay kit (R&D Systems, USA) according to the manufacturer's instructions. Briefly, standard or sample was added into per well and incubated for 1 hour. Subsequently, wash buffer, conjugate, substrate solution, and stop solution were added according to the instruction. Finally, we used a microplate reader to determine the optical density. Other biochemical tests were all performed using routine clinical autoanalyser assays in the Biochemistry Department of Zhongshan Hospital, including NT-proBNP, serum cholesterol, triglycerides, alanine aminotransferase, urea nitrogen, and creatinine.

### 2.3. Power Calculation

We were unaware of previous studies assessing the cross-sectional differences in ANG between CHD patients complicating CHF or not to power our study. It had been previously reported that ANG levels in heart failure exceeded those of healthy controls [6]; we therefore suggested similar difference in our enrolled patients. To achieve this similar increase at *p* < 0.05, 1 − *β* power of 0.90, and ratio of disease controls to CHF patients taking 2 : 1, a minimum of 89 disease controls and 45 CHF patients were required.

### 2.4. Statistical Analysis

Continuous variables were tested for normal distribution by Kolmogorov-Smirnov test and appropriately presented as mean (SD) or median (IQR, interquartile range). Categorical variables were shown as percentage. Comparisons between groups were performed by one-way ANOVA (or Kruskal-Wallis test), as appropriate, with Bonferroni post hoc test for intergroup comparisons. Comparison between categorical data were using the chi-square test or a Fisher's exact test. Correlations between two continuous variables were performed using the Pearson correlation coefficient, or Spearman rank correlation for nonnormally distributed data. Ordinal regression analysis was used to determine changes in ANG levels in relation to disease severity, where the pseudo *R*-square value was reported to explain the variation in ANG in response to the severity of disease. Receiver operator characteristic (ROC) curves were used to evaluate the performance of ANG, NT-proBNP, and LVEF, depicted by the mean area under the curve (AUC) with 95% CI. We treated *p* values < 0.05 as a statistically significant and used Stata 12.0 for Windows (StataCorp, TX, USA) to perform statistical analysis.

## 3. Results

### 3.1. Patients

**Figure 1:**
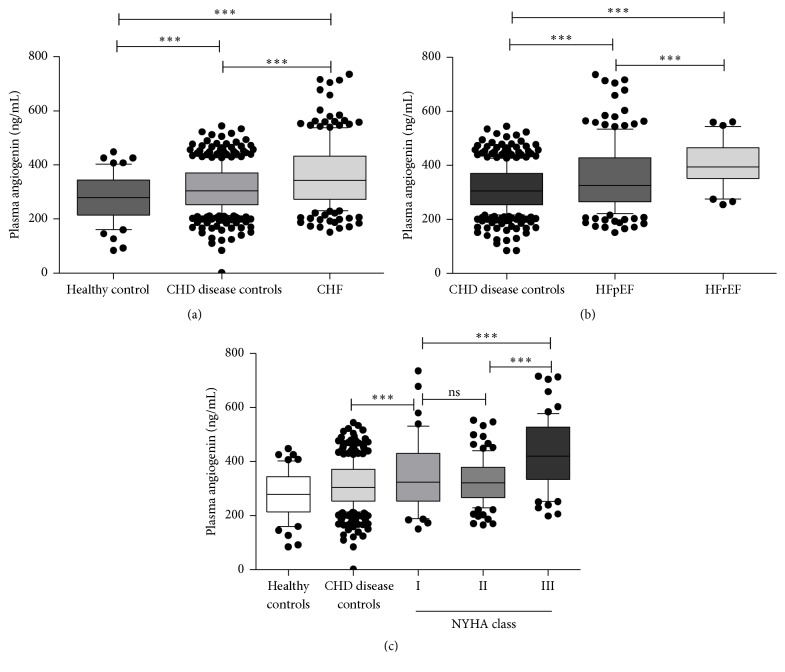
Plasma ANG levels in different groups. Box-and-whiskers plots (whiskers: 10–90 percentile). CHF: chronic heart failure. HFpEF: heart failure with preserved ejection fraction. HFrEF: heart failure with reduced ejection fraction. NYHA class: New York Heart Association class. ^*∗∗∗*^ *p* < 0.001.

We recruited 616 CHD patients, in which 203 ones complicating CHF were set as CHF group and the other 413 CHD patients as CHD disease controls. Additionally, 53 healthy subjects were recruited as healthy controls (Table 1). There were no differences in age, gender, hypertension, diabetes mellitus, dyslipidemia, or myocardial infarction between the three groups. And the ALT, BUN, and TG present no difference in these three groups. However, the FPG and TC were higher in the groups of CHF and disease controls than in healthy controls. NT-proBNP and LVEF did not show significant difference between the disease and healthy controls, while in the CHF group they were, respectively, elevated and decreased dramatically. Interestingly, the plasma levels of ANG were not only elevated substantially in CHF group, but also significantly higher in disease controls compared with healthy controls (Figure 1(a)).

### 3.2. ANG Elevated with the Exacerbation of CHF

Among CHF patients, plasma ANG levels showed no difference between different gender and those with or without MI history, hypertension, dyslipidemia, or diabetes mellitus status. Concerning the NYHA class, no difference of plasma ANG levels was observed between the NYHA I and NYHA II, but it elevated dramatically in the NYHA III-VI patients. (Figure 1(c)). On ordinal regression analysis, plasma ANG levels increased with deteriorating cardiac function (pseudo *R* ^2^ = 0.0397, *p* < 0.001).

With regard to observing disparity of plasma ANG levels between HFpEF (LVEF ≥ 0.50) and HFrEF (LVEF ≤ 0.40) group, we performed subgroup analysis of these two groups according to the guideline's LVEF classification criteria [1]. In HFrEF group, plasma ANG levels were substantially higher compared with HFpEF group (393.6 (IQR [351.1,464.9]) versus 322.8 (IQR [262.7,417.2]) ng/mL, *p* < 0.001), while its levels in HFpEF group were higher than CHD disease controls significantly (322.8 (IQR [262.7,417.2]) versus 304.5 (IQR [254.0,370.5]) ng/mL, *p* < 0.001) (Figure 1(b)). However, there was no difference of plasma angiogenin levels between HFpEF and borderline HFpEF (LVEF = 0.41–0.49) (322.8 (IQR [262.7,417.2]) versus 342.5 (IQR [277.3,432.6]) ng/mL) (Table 2).

### 3.3. Univariate Analysis

On bivariate analysis of patients and controls, levels of ANG were positively associated with fasting plasma glucose, deteriorating cardiac function (BNP, LVEF), and blood lipids levels (cholesterol, triglycerides), while it had no association with age (Table 3). In relation to cardiac function of CHF patients, ANG levels were negatively associated with LVEF (Spearman's rho = −0.2233, *p* < 0.01) and positively correlated with NT-proBNP (Spearman's rho = 0.3224, *p* < 0.001) among CHF patients.

### 3.4. Diagnostic Value of ANG in CHF and HFpEF

**Figure 2:**
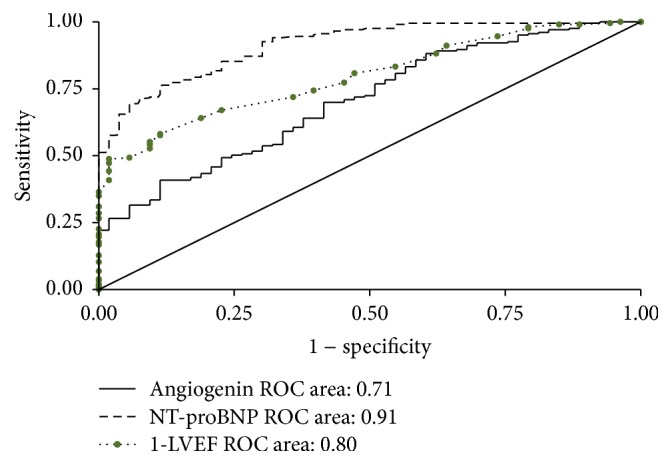
Receiver operating characteristic (ROC) curve of the diagnosis ability of ANG, NT-proBNP, and LVEF. LVEF: left ventricular ejection fraction.

With respect to the presence of CHF across disease and healthy controls, we detected receiver operating characteristics (ROC) of ANG, which showed ANG was a significant but not a good discriminator (AUC 0.71, 95% CI 0.63–0.78, *p* < 0.001), with a poorer performance compared to LVEF (AUC 0.80, 95% CI 0.74–0.85, *p* < 0.001) or NT-proBNP levels (0.91, 95% CI 0.87–0.95, *p* < 0.001) (Figure 2). Concerning ANG discriminative value for HFpEF and controls (disease and healthy), the AUC was even minor (0.59, 95% CI 0.54–0.64), while the AUC of NT-proBNP and LVEF were 0.86 (95% CI 0.82–0.89) and 0.7499 (95% CI 0.71–0.79), respectively.

## 4. Discussion

Chronic heart failure (CHF) is a major health problem worldwide and requires frequent readmissions to the hospital. Coronary heart disease (CHD) constitutes the major underlying causes of CHF and its mortality rates in general have declined for the past 25 years in most Western countries. Remarkable changes have taken place in the factors that contribute to the incidence of CHD complicating CHF, but the quantity of these patients is still large [10, 12].

The cornerstone of diagnosis of heart failure is a comprehensive history and physical examination, combining laboratory testing including NT-proBNP and echocardiography. However, NT-proBNP reflects myocardial wall tension and increases with increasing age [13]. And echocardiography allows the assessment of left ventricular systolic and diastolic function, wall thickness, ventricular dilation, and regional wall motion abnormalities provide evidence of the underlying etiology and chronicity of heart failure. Meanwhile, a number of additional biomarkers characterize inflammation, myocyte injury, neurohormonal upregulation, and extracellular matrix turnover in patients with heart failure, which may facilitate risk stratification to guide clinical judgment. In the CHD patients, cardiac remodelling is accompanied with angiogenesis; herein the angiogenic molecules may be a biomarker and indicate severity of this cohort.

ANG is a 14 124 Da soluble protein member of the ribonuclease (RNase) superfamily-enzymes, and its pathophysiological role in angiogenesis is what most studies focused on [14]. It has been common consensus that ANG is one of the most potent proangiogenic factors. Plasma ANG may be a biomarker for diseases in which angiogenesis is involved in the pathophysiology. ANG is elevated in various cancer types and some stress conditions such as inflammation and its plasma levels also increase [15–18]. In the territory of cardiology, ANG is elevated in acute coronary syndrome and predicts adverse events [4]. Another study has shown a series of angiogenesis markers including ANG progressive increase with hemodynamic and functional decline in CHF, while the patients included are majorly diagnosed with idiopathic cardiomyopathy [8].

In this study, we demonstrated ANG was elevated in the CHF patients compared with CHD patients or healthy controls. Furthermore, its level increased with the exacerbation of CHF, was higher in HFrEF than that in HFpEF patients, and was higher in NYHA class III-IV than that of class I or II. Meanwhile, plasma ANG levels in CHD patients were higher than that in healthy controls. However, ANG failed to be a good discriminator to identify CHF from the controls. All patients we enrolled were in stable phase coronary heart disease, and our results were in agreement with other studies where ANG might be a biomarker for heart failure [6, 19]. Therefore, elevated plasma ANG levels might imply the severity of cardiac remodelling in the stable phase CHD, especially in the CHF complicating CHD patients.

Our pilot study has found serum ANG elevated in HFpEF patients compared with either hypertension or healthy controls using antibody microarray and validated in a relative small cohort [7]. It is promising that ANG could be a biomarker to diagnose HFpEF [7]. However, in this study, we expanded the sample size, and the included CHF patients are all complicating CHD which is one of the most common CHF complicating diseases.

Although plasma ANG levels showed poorer diagnostic capacity in comparison with NT-proBNP or LVEF and undefined predictive value for the lack of follow-up, it positively correlated with NT-proBNP and LVEF which imply increased severity and poor prognosis of CHF [1]. Meanwhile, in other studies, elevated ANG predicted poor prognosis [4, 7]. Herein, ANG may be a predictor of the severity and prognosis of the CHD patients complicating CHF which needs further investigation.

The limitation of this study is cross-sectional approach, so that it is difficult to detect ANG's role in the pathophysiology of CHF or CHD in this study. For the absence of follow-up data, it is hard to determine ANG's predictive role in prognosis of CHF patients complicating CHD, although our prior study showed angiogenin may predict all-cause death in HFpEF. In this study, we set relative loose inclusion standard, which is in accordance with the reality better. However, the data showed ANG may be a moderate discriminator of CHF from CHD or the healthy.

In conclusion, ANG is elevated in CHD patients compared with healthy controls, and it is higher in the CHD patients complicating CHF, both the HFpEF and HFrEF, particularly in HFrEF subgroup. It may play a potent role in the progression of heart failure which needs further research.

## Figures and Tables

**Table 1:** Clinical characteristics and plasma ANG levels of patients and controls included in the study.

	CHF	CHD disease control	Healthy control
*N*	203	413	53
Age	65 ± 10	64 ± 10	63 ± 12
Male sex	159 (78.3%)	308 (74.6%)	36 (67.9%)
Hypertension	129 (63.5%)	284 (68.8%)	None
Diabetes mellitus	71 (35.0%)	133 (32.2%)	None
Dyslipidemia	18 (8.9%)	37 (9.0%)	None
Myocardial infarction	69 (34.0%)	128 (31.0%)	None
Angina	72 (35.5%)	170 (41.2%)	None
ALT (U/I)	19 (13,27.25)	20 (13,29)	20.5 (11.75,28.25)
BUN (*μ*mol/L)	5.8 (4.7,7.3)	5.4 (4.6,6.6)	5.3 (4.5,6.1)
FPG	5.6 (4.9,6.6)^*∗*^	5.4 (4.9,6.2)^*∗*^	4.7 (4.3,5.8)
TC (mmol/L)	3.8 (3.2,4.7)^*∗*^	3.8 (3.2,4.6)^*∗*^	3.7 (2.6,4.4)
TG (mmol/L)	1.4 (1.1,1.9)	1.4 (1.0,2.0)	1.2 (0.9,2.0)
hs-CRP	1.7 (0.9,3.9)	1.6 (0.8,4.0)	1.3 (0.6,1.5)
NT-proBNP (pg/mL)	747 (309.6,1617)^*∗*#^	134.1 (63.4,302.4)	121 (60.15,250.25)
LVEF (%)	55 (45,64)^*∗*#^	66 (62,70)	66 (66,70)
LAD (mm)	40.0 ± 6.3^*∗*^	39.0 ± 4.7	37.0 ± 5.4
LVEDD (mm)	53 (47,58)^*∗*#^	48 (44,51.8)	48.5 (44.3,51.8)
LVESD (mm)	35 (30,44)^*∗*#^	30 (27,33)	30 (28.34)
ANG (ng/mL)	342.8 (273.9,432.9)^*∗*#^	304.5 (254.0,370.5)^*∗*^	279.7 (214.4,344.0)

^*∗*^ *p* < 0.05 compared with healthy controls.

^#^ *p* < 0.05 compared with CHD disease controls.

CHD: coronary heart disease; CHF: chronic heart failure; LT: alanine aminotransferase; BUN: blood urea nitrogen; FPG: fasting plasma glucose; TC: total cholesterol; TG: triglyceride. NT-proBNP: N-terminal pro-B-type natriuretic peptide; LVEF: left ventricular ejection fraction; LAD: left atrial diameter; LVEDD: left ventricular end-diastolic dimension; LVESD: left ventricular end-systolic dimension; values are expressed as mean ± SD, as median (IQR), or as indicated.

**Table 2:** ANG levels in the CHF group.

ANG (ng/mL)	*n*	Median	Interquartile range	
Gender				
Female	44	344.6	273.5	402.2
Male	159	341.5	274.3	451.3
History of myocardial infarction				
Yes	69	341.5	273.9	432.3
No	134	344.5	273.3	435.6
History of hypertension				
Yes	129	354.9	289.8	452.0
No	74	325.0	260.2	404.3
History of dyslipidemia				
Yes	18	335.9	284.1	413.7
No	185	342.8	273.7	434.7
History of diabetes				
Yes	71	355.6	298.0	444.7
No	132	333.8	265.7	422.4
NYHA classification				
I	46	311.4	248.8	421.3
II	95	324.8	273.5	381.3
III-VI^*∗*^	62	420.5	333.6	527.0
LVEF				
≥0.5	129	322.8	262.7	417.2
0.41–0.49	40	342.5	277.3	432.6
≤0.4^#^	34	393.6	351.1	464.9

^*∗*^ *p* < 0.001 compared with NYHA I or II.

^#^ *p* < 0.001 compared with LVEF ≥ 0.5 or 0.41–0.49.

CHF: chronic heart failure; NYHA: New York Heart Association; LVEF: left ventricular ejection fraction.

**Table 3:** Correlation between ANG levels and cardiac risk markers in the patients and controls included.

Association with levels of plasma ANG (ng/mL)	Spearman's correlation coefficient	Sig. (two-tailed)
Age (years)	−0.0103	0.7900
Fasting plasma glucose (mmol/L)	0.0948	0.0143
Serum cholesterol (mmol/L)	0.0683	0.0775
Serum triglycerides (mmol/L)	0.2045	<0.001
hs-CRP	0.1257	0.0103
NT-proBNP (pmol/L)	0.2818	<0.001
LVEF	−0.2223	<0.001

NT-proBNP: N-terminal pro-B-type natriuretic peptide.

LVEF: left ventricular ejection fraction.

## Abbreviations

ANG: Angiogenin
CHD: Coronary heart disease
CHF: Chronic heart failure
HFrEF: Heart failure with reduced ejection fraction
HFpEF: Heart failure with preserved ejection fraction
LVEF: Left ventricular ejection fraction.
